# Plasma HSPA12B Is a Potential Predictor for Poor Outcome in Severe Sepsis

**DOI:** 10.1371/journal.pone.0101215

**Published:** 2014-06-30

**Authors:** Ran Zhang, Xiao-jian Wan, Xu Zhang, Qiu-xiang Kang, Jin-jun Bian, Gui-fang Yu, Jia-feng Wang, Ke-ming Zhu

**Affiliations:** 1 Department of Anesthesiology and Intensive Care Medicine, Changhai Hospital, the Second Military Medical University, Shanghai, China; 2 Department of Anesthesiology, the Third People’s Hospital, Shanghai Jiaotong University, Shanghai, China; University of Torino, Italy

## Abstract

**Introduction:**

Endothelium-derived molecules may be predictive to organ injury. Heat shock protein (HSP) A12B is mainly located in endothelial cells, which can be detected in the plasma of septic patients. Whether it is correlated with prognosis of sepsis remains unclear.

**Methods:**

Extracellular HSPA12B (eHSPA12B) was determined in plasma of septic mice at 6h, 12h, 24h and 48h after cecal ligation and puncture (CLP). It was also detected in plasma of patients with severe sepsis, sepsis, systemic inflammatory response syndrome and healthy volunteers. The predictive value for prognosis of severe sepsis was assessed by receiver operating curve (ROC) and Cox regression analyses.

**Results:**

eHSPA12B was elevated in plasma of CLP mice at 6h and peaked at 24h after surgery. A total of 118 subjects were included in the clinical section, including 66 patients with severe sepsis, 21 patients with sepsis, 16 patients with SIRS and 15 volunteers. Plasma eHSPA12B was significantly higher in patients with severe sepsis than in patients with sepsis, SIRS and volunteers. The level of eHSPA12B was also higher in non-survivals than survivals with severe sepsis. The area under the curve (AUC) of eHSPA12B in predicting death among patients with severe sepsis was 0.782 (0.654–0.909) in ROC analysis, much higher than that of IL-6 and IL-10. Cox regression analysis showed that cardiovascular diseases, IL-6 and eHSPA12B were risk factors for mortality in patients with severe sepsis. Survival curve demonstrated a strikingly significant difference between 28-day survival rates of patients with an eHSPA12B lower or not lower than 1.466ng/ml.

**Conclusions:**

Plasma eHSPA12B is elevated in both septic mice and patients. It may be a good predictor for poor outcome in patients with severe sepsis.

## Introduction

Sepsis has been well recognized as a systemic inflammatory response to an active infection process, which is still a common cause of mortality in intensive care units (ICU). Severe sepsis, a worse form of sepsis, is characterized by sepsis-induced organ failure. A well-designed survey in America reported that incidence of sepsis was 1.3% of all hospitalizations and the mortality was 17.9% in 2000 [1]. Mortality in patients with severe sepsis was reported to be over 40% in the epidemiological studies from different countries over the world, much higher than in septic patients without organ dysfunction [2]–[6]. Early identification of septic patients with poor outcome is generally considered as a critical barrier to deliver optimal management including monitoring, fluid resuscitation and antibiotic treatment [7]. Surviving sepsis campaign guidelines suggested that early administration of antibiotics should be administrated within the first hour of recognition of severe sepsis and septic shock [8]. Early, monitored management was reported to reduce 30-day mortality by 26% in patients with severe sepsis according to a prospective before and after study [9].

Several biomarkers have been developed for early identification of high-risk patients with severe sepsis, including numerous chemokines, cytokines and procalcitonin (PCT) [10]–[11]. Recently, biomarkers for endothelial injury, such as von Willebrand factor (vWF), soluble intercellular adhesion molecule-1 (sICAM-1), endothelial protein C and angiopoietins, are becoming promising biomarkers for severe sepsis [12]–[14], because endothelial dysfunction is widespread in company with organ dysfunction [15]. Guitton et al. [16] found that transient increase of endothelial protein C was associated with poor outcome in severe sepsis. Ricciuto et al. [14] reported that angiopoietin-1 and angiopoietin-2 were correlated with increased mortality and the latter was associated with organ dysfunction, which suggested that they might serve as informative biomarkers for severe sepsis.

Heat shock protein (HSP) A12B, the newest member of HSP-70 family, is mainly located in endothelial cells [17]. Its presence is essential for angiogenesis and endothelial functions in different species [18]. Overexpression of HSPA12B has been demonstrated to be benefit for endotoxin-induced cardiac dysfunction and cerebral ischemia reperfusion injury in mice [19], [20]. HSP was reported to be present in plasma and serum to trigger innate immunity and serve as biomarker for diseases. For example, HSP70 was reported to be up-regulated in plasma of patients with chronic myeloid leukemia and sarcopenia and might be a potential biomarker for these diseases [21], [22]. It was unknown whether HSPA12B existed in the plasma of patients with endothelial injury. The correlation of extracellular HSPA12B (eHSPA12B) with organ dysfunction was unclear, but it might be a promising biomarker for endothelial injury because of its initial location, primarily in endothelial cells. Therefore, this present study was performed to detect eHSPA12B level in plasma of septic mice and patients with sepsis and severe sepsis. Its correlation with the outcome was also evaluated to determine the potential role in predicting prognosis.

## Patients, Materials and Methods

### Cecal ligation and puncture model

The animal study was approved by the Animal Care and Use Committee of Changhai Hospital (Shanghai, China). The Cecal ligation and puncture (CLP) procedure was carried out as previously described [23] to detect the presence of eHSPA12B in the plasma as well as the dynamic changes of plasma eHSPA12B at 6h, 12h, 24h and 48h. Six- to eight-week C57BL/6J mice (n = 24) were included in the experiments. After anaesthetized by inhalation with 3% sevoflurane (Baxter, USA), the cecum was exposed through a 1.5 cm longitudinal incision in lower quadrants of the abdomen. The distal three-fourth of the cecum was ligated with a 4–0 silk suture and punctured for twice with a 22-gauge needle. The cecum was then replaced into peritoneal cavity and the incision was closed by suturing in 2 layers. Sham-operated mice underwent the same procedure, but without ligation and needle perforation on the cecum. After surgery, the mice were injected subcutaneously with 1 ml of sterile saline and allowed for free access to food and water after awaking. For dynamic analysis of eHSPA12B level in plasma, blood sample was collected by heart puncture at 6h (n = 6), 12h (n = 6), 24h (n = 6) and 48h (n = 6) after surgery, respectively. For the correlation analysis of eHSPA12B with disease severity, plasma eHSPA12B level was compared among the sham-operated mice, mice punctured one and twice in the cecum. The relationship between eHSPA12B and IL-6 was also analyzed to further evaluate the correlation of eHSPA12B with disease severity.

### Design of the clinical trial

A prospective case-control study was performed in this part, recruiting patients with severe sepsis, simple sepsis without organ dysfunction and systemic inflammatory response syndrome (SIRS), as well as healthy volunteers. The study protocol was reviewed and approved by the ethical committee of the Second Military Medical University. Written informed consent was obtained from all subjects or their lineal relations. The trial was registered in Clinicaltrial.gov with an ID of NCT01847248.

### Patients

Adult patients were recruited consecutively from May 2011 to October 2013 in Intensive Care Unit of Changhai Hospital. The diagnosis of sepsis and severe sepsis was based on 2001 SCCM/ESICM/ACCP/ATS/SIS International Sepsis Definitions Conference [24]. Generally, sepsis was diagnosed by an identifiable or suspected infection site and evidence of SIRS manifested by at least two of the following criteria: 1) body temperature >38°C or <36°C; 2) heart rate >90 beats/min; 3) respiratory rate >20 breaths/min; 4) a white blood cell count >12,000/mm^3^ or <4,000/mm^3^. Severe sepsis was diagnosed when septic patient suffered from at least one organ dysfunction within 24h hours after inclusion. In this study, patients with sepsis but not severe sepsis were defined as sepsis group. A total of 16 patients undergoing major orthopedics surgery were included in a SIRS group, and 15 healthy volunteers were included in a control group. The exclusion criteria included: 1) patients younger than 18 years old; 2) patients without informed consent; 3) patients undergoing continuous renal replacement therapy before sampling; 4) patients receiving immunosuppressive or steroid therapy; 5) patients with special infection induced by virus, tubercle bacillus, mycoplasma, Chlamydia, and so on. blood samples were collected by venous puncture and stored in BD Vacutainer tubes with lithium heparin (BD, NJ, US). Blood samples from patients with sepsis, severe sepsis and SIRS were collected within 24h after onset of sepsis or SIRS. In order to observe the dynamic changes of plasma eHSPA12B, blood samples were collected at 1d, 3d and 5d after onset of sepsis. All the samples were frozen at −80°C after centrifugation.

### Data collections

The general data included demographic characteristics, primary diagnosis, co-morbidities, infection sites and the pathogen type of causative micro-organisms. Co-morbidities included pre-existing diabetes mellitus and cardiovascular diseases (hypertension and coronary heart disease). Outcome included 28-day mortality, duration of ventilation, length of stay (LOS) in ICU, and severity scores such as the Acute Physiology and Chronic Health Evaluation (APACHE) II score, the Sequential Organ Failure Assessment (SOFA) score, and Marshall’s Multiple Organ Dysfunction Syndrome (MODS) score.

### Sampling and enzyme linked immunosorbent assay

Levels of plasma eHSPA12B were determined by enzyme-linked immunosorbent assay (ELISA), as well as interleukin-10 (IL-10) and interleukin-6 (IL-6) as control. But IL-6 and IL-10 were just detected in patients with sepsis or severe sepsis. All of the three proteins were determined using a commercial solid-phase ELISA kit according to the manufacturer’s instructions (CUSBIO, Wuhan, China). The detection limit of HSPA12B, interleukin-10 (IL-10) and interleukin-6 (IL-6) was 0.312 ng/ml, 12.5 pg/ml, and 7.8 pg/ml, respectively. All ELISA assays were performed by an investigator who was blinded to the groups.

### Statistical analysis

Continuous variables are presented as mean±SD or median (with interquartile range) (IQR), and categorical variables as numbers and percentages. Two-group was compared with unpaired Student’s *t*-test or Mann-Whitney U test for continuous variables and chi-square test for categorical variables. For multi-group comparison, Kruscal-Wallis test was used with Bonferroni post hoc evaluation. Correlations between two continuous variables were assessed by the Pearson’s correlation analysis. Receiver operating characteristic (ROC) curves were utilized to evaluate the accuracy of eHSPA12B, IL-6 and IL-10 to diagnose sepsis, severe sepsis or prognosis. Survival rate was compared using Kaplan-Meier tests, and determinants correlated with prognosis were analyzed by Cox regression analysis. All statistical analysis was performed in SPSS 16.0 software (SPSS Inc., IL, USA). *p*<0.05 was regarded as statistically significant.

## Results

### Plasma eHSPA12B in CLP mice

**Figure 1:**
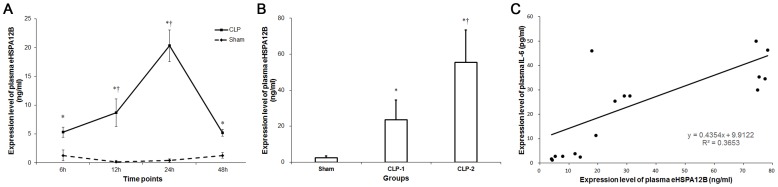
Expression level of eHSPA12B in the plasma of septic mice. A. Plasma levels of eHSPA12B in septic mice. eHSPA12B was elevated at 6(n = 6 for each time points of the 2 groups). * *p*<0.001, compared with sham group; † *p*<0.001, compared with the level at 6h. Data analyses with Student’s *t*-test. B. Plasma level of eHSPA12B in mice with sepsis of different severity. CLP-1, mice with once puncture in the cecum; CLP-2, mice with twice punctures in the cecum. * *p*<0.001, compared with sham group; † *p*<0.001, compared with CLP-1 group. Data analyses with ANOVA test. C. Relationship between the levels of eHSPA12B and IL-6 (*p*<0.001). Data analyses with Pearson’s correlation analysis.

As shown in Figure 1A, levels of plasma eHSPA12B in CLP mice was significantly higher at all time points from 6h to 48h after CLP surgery than in Sham-operated mice (*p*<0.05). With the progression of sepsis, plasma eHSPA12B was elevated and peaked at 24h, followed by decreasing to a level at 48h similarly to that at 6h. As shown in Figure 1B, plasma eHSPA12B was elevated with the increasing severity. Mice with twice punctures in the cecum had the highest level of plasma eHSPA12B. The plasma eHSPA12B was also linearly correlated with the IL-6 level, which was a marker of sepsis severity [25] (Figure 1C).

### Patient characteristics

In order to further clarify the clinical significance of circulating eHSPA12B in septic patients, a total of 118 patients were recruited in the case-control study, including 66 patients with severe sepsis, 21 patients with sepsis, 16 patients with simple SIRS and 15 volunteers. The general data of these patients was shown in Table 1. Septic patients were significantly older than SIRS patients and volunteers (*p*<0.001), while the ages were similar between patients with severe sepsis and simple sepsis, as well as between SIRS patients and volunteers. Gender were comparable among the 4 groups, while patients in sepsis group tended to have more co-morbidity of diabetes mellitus than in severe sepsis and SIRS groups (*p = *0.047). There were more patients underwent major abdominal surgery and less patients with lithangiuria in severe sepsis group (*p* = 0.028). There were no significant differences regarding Infection sites and pathogen types (*p*>0.05). Severity scoring was significantly higher in patients with severe sepsis, among whom duration of ventilation and LOS in ICU were also longer (*p*<0.001). No patient was dead in sepsis group, while 26 patients died in severe sepsis group (*p*<0.001).

**Table 1:** General data of subjects.

Variables	Sepsis (n = 21)	severe sepsis (n = 66)	SIRS (n = 16)	Volunteers (n = 15)	*p*
Age(Mean ± SD)	58.2±16.4	63.7±14.9	51.1±11.6	44.3±17.4	<0.001
Male/Female	12/9	48/18	8/8	8/7	0.193
Co-morbidity [No. (%)]					
cardiovascular disease	13 (61.9)	27 (40.9)	4 (25)	–	0.071
diabetes mellitus	9 (42.9)	22 (33.3)	1 (6.25)	–	0.047
Primary diagnosis [No. (%)]					0.028
major abdominal surgery	5 (23.8)	36 (54.5)	–	–	
intestinal obstruction	3 (14.3)	8 (12.1)	–	–	
intestinal perforation	5 (23.8)	10 (15.2)	–	–	
lithangiuria	7 (33.3)	2 (3.0)	–	–	
others	1 (4.8)	10 (15.2)	–	–	
Infection site [No. (%)]					0.493
abdomen	14 (66.7)	50 (75.8)	–	–	
respiratory	0 (0)	5 (7.6)	–	–	
urine	6 (28.6)	2 (3.0)	–	–	
blood	1 (4.7)	7 (10.6)	–	–	
mixed	0 (0)	2 (3.0)	–	–	
Pathogen type [No. (%)]					0.533
Gram-negative	10 (47.6)	21 (31.8)	–	–	
Gram-positive	4 (19)	5 (7.6)	–	–	
Fungus	1 (4.8)	0 (0)	–	–	
Mixed	3 (14.3)	25 (37.9)	–	–	
APACHE II score [median (IQR)]	8.0 (6.0, 11.5)	18.5 (14.8, 23.0)	–	–	<0.001
SOFA score [median (IQR)]	1.0 (0, 2.0)	7.0 (4.0, 11.0)	–	–	<0.001
MODS score [median (IQR)]	0 (0, 1.5)	5.5 (3.0, 7.0)	–	–	<0.001
Duration of MV [median (IQR)]	2.0 (1.0, 2.5)	5.0 (2.0, 12.0)	1 (1, 2)	–	<0.001
ICU stay [median (IQR)]	2.0 (1.0, 5.0)	9.5 (3.0, 18.0)	2 (2, 3)	–	<0.001
28-day mortality No.(%)	0 (0)	26 (39.4)	–	–	<0.001

*SD* standard deviation, *IQR* inter-quartile range, *APACHE II* Acute Physiology and Chronic Health Evaluation II, *SOFA* Sequential Organ Failure Assessment, *MODS* Multiple Organ Dysfunction Syndrome, *MV* mechanical ventilation, *ICU* intensive care unit.

### Level of eHSPA12B, IL-6 and IL-10 in plasma

**Figure 2:**
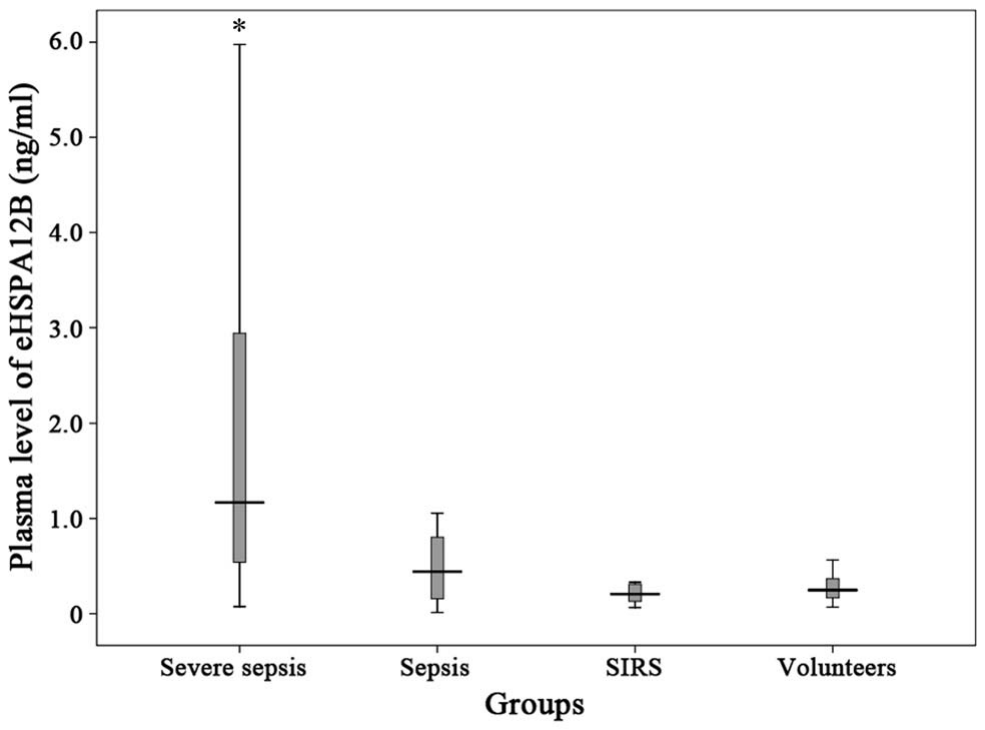
Plasma levels of eHSPA12B in patients. Subjects included patients with severe sepsis (n = 66), sepsis (n = 21), SIRS (n = 16) and healthy volunteers (n = 15). eHSPA12B was significantly higher in patients with severe sepsis than those in other 3 groups. * *p*<0.001, compared with the other 3 groups. Data analyses with Mann-Whitney U test.

**Figure 3:**
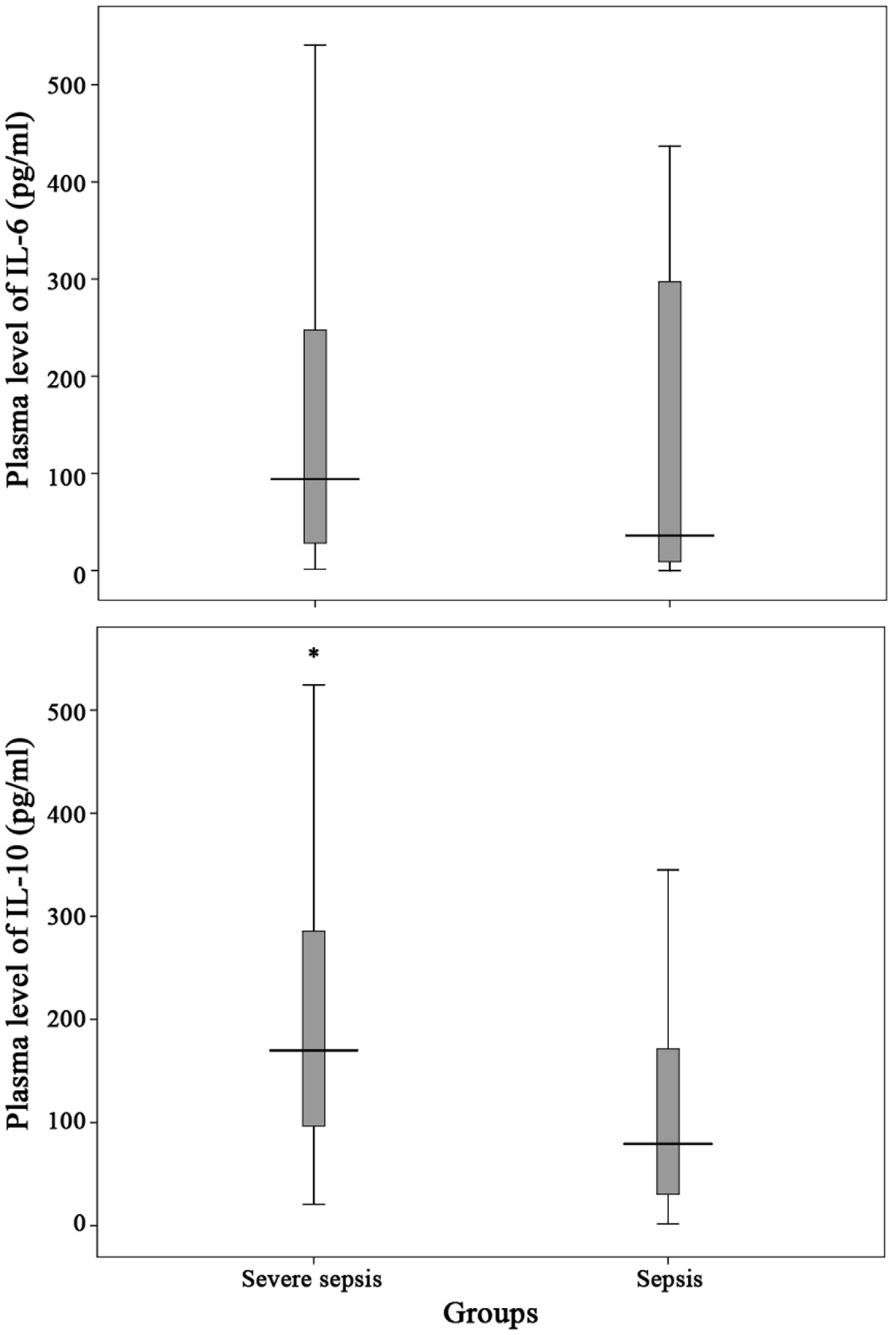
Plasma level of IL-6 and IL-10 in patients with severe sepsis and sepsis. Patients with severe sepsis had slightly higher plasma IL-10 level but similar IL-6 level when compared with those with sepsis. * *p* = 0.009, compared with sepsis group. Data analyses with Mann-Whitney U test.

The level of plasma eHSPA12B was significantly higher in patients with severe sepsis or septic shock than other three groups (*p<*0.001) (Figure 2). The level of eHSPA12B in patients with sepsis was slightly higher than SIRS patients and healthy volunteers without significant difference (*p* = 0.294 and *p* = 0.391). No differences were observed between SIRS patients and healthy volunteers (*p* = 0.446). IL-10 level was significantly higher in severe sepsis group than in sepsis group (*p* = 0.009), while no significant difference was found when comparing IL-6 level between these 2 groups (*p* = 0.115) (Figure 3).

### Predictive value of eHSPA12B, IL-6 and IL-10 for prognosis in patients with severe sepsis

The relationship between eHSPA12B and prognosis of severe sepsis was analyzed in comparison with IL-6 and IL-10. Patients were divided into survivals and non-survivals according to the 28-day mortality, and the demographic characteristics were compared between survival and non-survivals in Table 2. Incidence of pre-existing cardiovascular disease was higher in non-survivors than in survivors (*p* = 0.001). Non-survivors had higher APACHE II score and plasma levels of IL-6, IL-10 as well as eHSPA12B (*p*<0.05) (Table 2).

**Table 2:** Demographic comparison between survivors and non-survivors with sepsis or severe sepsis.

Variables	Survivor (n = 40)	Non-survivor (n = 26)	*p*
Age (Mean ± SD)	62.6±14.8	65.5±15.3	0.443
Male/Female	41584	41407	0.632
Co-morbidity [No. (%)]			
cardiovascular disease	10 (25.0)	17 (65.4)	0.001
diabetes mellitus	12 (30.0)	10 (38.4)	0.476
Primary diagnosis [No. (%)]			0.713
major abdominal surgery	21 (52.5)	15 (57.7)	
intestinal obstruction	4 (10.0)	4 (15.4)	
intestinal perforation	6 (15.0)	4 (15.4)	
lithangiuria	2 (5.0)	0 (0)	
others	7 (17.5)	3 (11.5)	
Infection site [No. (%)]			0.377
abdomen	30 (75.0)	20 (76.9)	
respiratory	2 (5.0)	3 (11.5)	
urine	2 (5.0)	0 (0)	
blood	4 (10.0)	3 (11.5)	
mixed	2 (5.0)	0 (0)	
Pathogen type [No. (%)]			0.109
Gram-negative	12 (30.0)	3 (11.5)	
Gram-positive	13 (32.5)	8 (30.8)	
fungus	1 (2.5)	4 (15.4)	
mixed	14 (35.0)	11 (42.3)	
APACHE II score [median (IQR)]	16.5 (13.0, 20.8)	20.5 (16.0, 25.3)	0.015
SOFA score [median (IQR)]	6.0 (3.0, 9.8)	9.5 (5.5, 12.0)	0.069
MODS score [median (IQR)]	5.0 (2.0, 7.0)	6.0 (4.0, 7.3)	0.166
IL-6	69.6 (1.2, 180.5)	140.1 (6.9, 557.2)	0.036
IL-10	14.6 (8.5, 25.5)	24.3 (1.5, 32.4)	0.041
eHSPA12B	0.9 (0.5, 1.3)	2.9 (1.4, 6.7)	<0.001

*SD* standard deviation, *IQR* inter-quartile range, *APACHE II* Acute Physiology and Chronic Health Evaluation II, *SOFA* Sequential Organ Failure Assessment, *MODS* Multiple Organ Dysfunction Syndrome, *MV* mechanical ventilation, *ICU* intensive care unit, *IL-6* interleukin 6, *IL-10* interleukin 10, *eHSPA12B* extracellular heat shock protein A12B.

**Figure 4:**
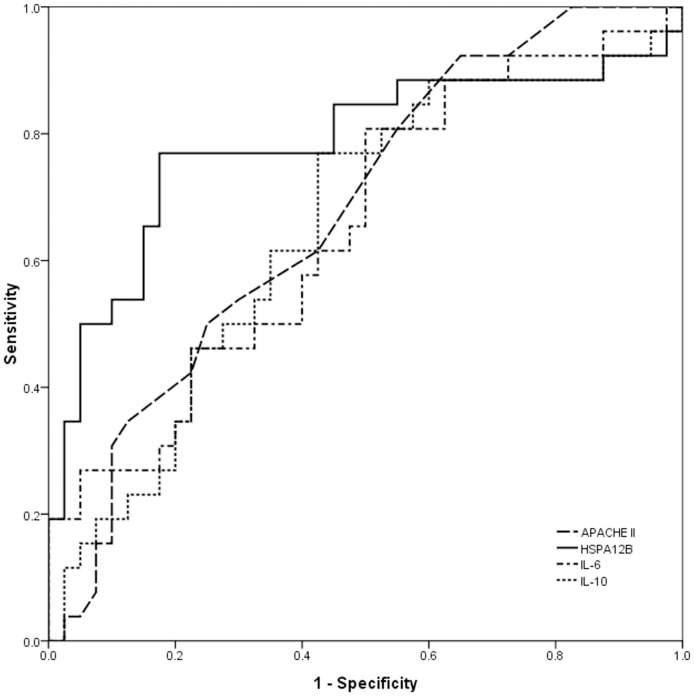
Receiver operator curve analysis of APACHE II score, eHSPA12B, IL-6 and IL-10, for predicting death in patients with severe sepsis. Area under the curve of HSPA12B was markedly higher than APACHE II score, IL-6 and IL-10. Data analyses with ROC curves.

Next, ROC analysis was performed to compare their predictive value for death in severe sepsis (Figure 4). The AUC results showed the areas of eHSPA12B [0.782 (0.654–0.909)] was much higher than of APACHE II score [0.678, (0.549, 0.807)], IL-6 [0.654 (0.518–0.789)] and IL-10 [0.650 (0.513–0.787)]. The optimal cut-off value of eHSPA12B was demonstrated to be 1.466/ml, with a sensitivity of 0.769 and a specificity of 0.825.

**Figure 5:**
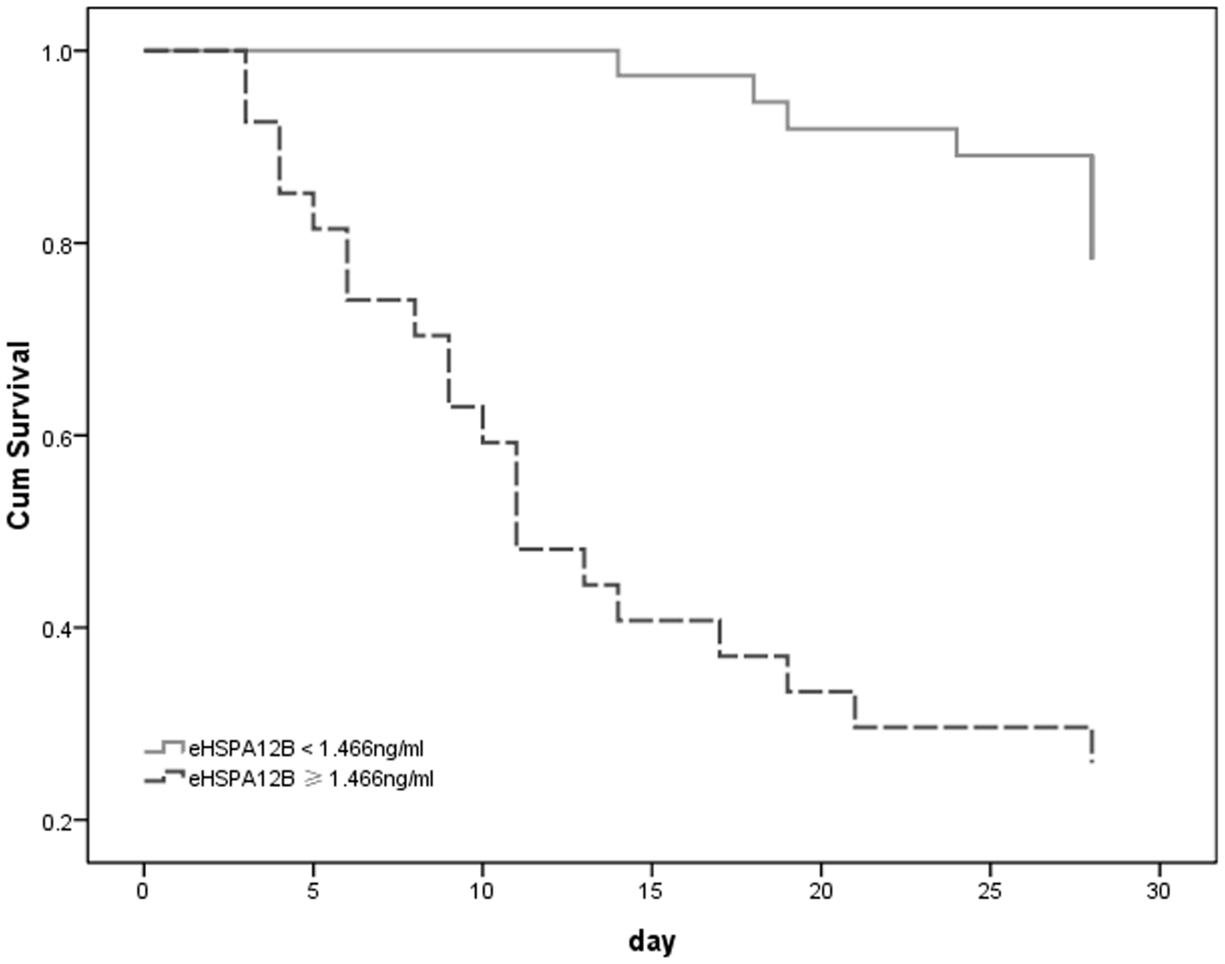
Survival analysis of patients with different levels of eHSPA12B. Mortality was significantly higher in patients with an eHSPA12B level not lower than 1.466ng/ml than in patients with an eHSPA12B level lower than 1.466ng/ml (*p*<0.001). Data analyses with Kaplan-Meier analysis.

Finally, variables in Table 2 with a *p* value lower than 0.1 were included in a Cox regression analysis model to identify the risk factors for prognosis of severe sepsis. Cox regression analysis showed that cardiovascular disease, IL-6 and eHSPA12B level were correlated with prognosis of patients with severe sepsis (Table 3). A further survival curve was generated to compare survival of patients with an eHSPA12B level lower or not lower than 1.466 ng/ml. The 28-day survival rates were strikingly lower in patients with an eHSPA12B level higher than 1.466 ng/ml (*p*<0.001) (Figure 5).

**Table 3:** Cox regression analysis for factors associated with prognosis of severe sepsis.

Variables	*p*	Hazard ratio	Hazard ratio (95% CI)	
			Lower	Upper
Cardiovascular disease	0.005	3.317	1.433	7.678
IL-6	0.004	1.002	1.001	1.003
eHSPA12B	<0.001	1.222	1.118	1.337

*CI* confidential index, *IL-6* interleukin 6, *eHSPA12B* extracellular heat shock protein A12B.

### Dynamic changes of eHSPA12B in patients with severe sepsis

**Figure 6:**
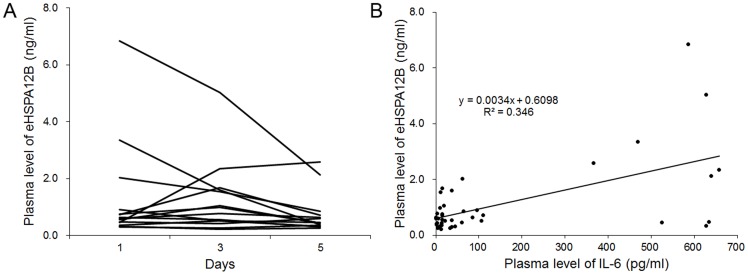
Time frame of plasma eHSPA12B in septic patients and its correlation with IL-6. A. Dynamic changes of plasma eHSPA12B in 14 patients at 1d, 3d and 5d after onset of sepsis. B. Correlation analysis between the levels of eHSPA12B and IL-6 (*p*<0.001). Data analyses with Pearson’s correlation analysis.

Dynamic changes of eHSPA12B was analyzed in 14 patients with severe sepsis at 1d, 3d and 5d after onset of sepsis. The 14 patients included 10 males and 4 females. None of them died within 7 days. The diagnosis included intestinal obstruction in 6 patients, intestinal perforation in 5 patients, and pneumonia in 2 patients. The APACHE II score was 11 (8, 19.75). As shown in Figure 6A, the dynamic changes of eHSPA12B varied greatly among different patients. Since IL-6 was higher in non-survivors, we further analyzed the relationship between IL-6 and eHSPA12B across the 3 days. As a result, eHSPA12B was linearly correlated with IL-6 (*p*<0.001) (Figure 6C).

## Discussions

The present study, for the first time, reported that eHSPA12B is specifically high expressed in plasma of septic mice and patients with severe sepsis. eHSPA12B level was higher in septic mice with higher severity. Its level was correlated with poor outcome in patients with severe sepsis, with high specificity and sensitivity to predict mortality. Patients with an eHSPA12B higher than 1.466/ml are at high risk of death.

Severe sepsis is life threatening with mortality higher than 40% as reported in several epidemiological studies from different continents from almost all over the world [2]–[6], [26]. Therapeutic bundles proposed by the Surviving Sepsis Campaign (SSC) are helpful to improve survival among patients with severe sepsis, and the mortality was reported to be reduced by 13% and 4.3% as reported by Lefrant et al. [27] and Suarez et al. [28] respectively. However, the SSC protocols were not well complied by in Europe and US, with a complete compliance ratio of just 18.4% and 21.6% [29]. According to the guidelines, early initiation of SSC protocols is crucial for surviving patients with severe sepsis or septic shock, especially within the first 6 hours [30]. Therefore, early recognition of high-risk patients with severe sepsis is extremely important for improving the therapeutic status of this fatal syndrome.

Fast-detected biomarkers have been focus by many investigators in the past decades and a wide variety of molecules or techniques are developed in purpose of diagnosing and predicting prognosis for sepsis or severe sepsis [10]–[11], [31]. C-reactive protein (CRP), tumor necrosis factor-α (TNF-α) and interleukins are among the first-discovered potential biomarkers for sepsis, among which IL-6 and IL-10 may be good choices as biomarkers while CRP and TNF-α are nonspecific biomarkers for inflammation [32], [33]. IL-6 and IL-10 has also been demonstrated to be associated with prognosis of severe sepsis [34], [35]. It was interesting to notice that IL-10 was higher in patients with severe sepsis, while IL-6 was similar between patients with severe sepsis and simple sepsis. But in the Cox regression, IL-10 was excluded while IL-6 was demonstrated to be correlated to the prognosis of patients with severe sepsis. Compared with IL-6, eHSPA12B was more suitable as a prognostic factor because of a much higher AUC in the ROC analysis. The surviving analysis further confirmed that mortality rate was much higher in patients with an eHSPA12B higher than 1.466/ml.

HSPA12B could be regarded as an endothelium-derived molecule [18], which might be a rational biomarker for organ dysfunction. Alteration of endothelial function is a central process of the pathogenesis of severe sepsis [15]. Firstly, activated endothelial cells by endotoxin or inflammatory mediators expose cell surface phospholipids and induce binding with coagulation complexes, which resulted in microcirculation dysfunction, tissue hypoxia and damage. Secondly, adhesion molecules are upregulated on endothelial cells in response to inflammation, such as selectins, intracellular and vascular cell adhesion molecules. Enhanced inflammatory cells adhesion and migration by them aggravated tissue damage and blockade of these adhesion molecules has been demonstrated to be protective against sepsis in vivo. Thirdly, endothelial injury and apoptosis increase vascular permeability. Loss of the barrier function leads to tissue edema and impaired organ function, which is the main feature of lung injury. Therefore, biomarkers for endothelial dysfunction may also be ideal markers for disease severity. Several endothelium-related molecules such as angiopoietins, vWF and sICAM-1 were demonstrated to be correlated with severity of sepsis [12]–[14], and similarly, HSPA12B, mainly located in endothelial cells, was considered as a predictor for prognosis of severe sepsis.

Our results also suggested that pre-existing cardiovascular diseases was a risk factor for mortality in patients with severe sepsis. It was reported that new-onset atrial fibrillation was an independent risk factor of stroke and death in patients with severe sepsis [36], [37]. Pre-existing cardiovascular diseases might induce the myocardial remodeling and predispose the patients to the development of new-onset atrial fibrillation. Chronic medical conditions such as peripheral artery disease, coronary heart disease and hypertension might also increase the risk of sepsis occurrence [38]. Therefore, cardiovascular diseases including coronary heart disease and hypertension should be regarded as an important risk factor for death among patients with severe sepsis.

Finally, the dynamic changes of eHSPA12B seemed to be absolutely different among most of patients. But the IL-6 level is a biomarker of sepsis severity, and our data also suggested that IL-6 was higher in non-survivors. Since eHSPA12B level at 1d, 3d and 5d after sepsis was correlated with IL-6, the dynamic changes of eHSPA12B might be correlated with the changes of disease severity.

### Limitations

Several limitations are present in our present study. Sample size is relatively small for a biomarker study. However, the circulating level of eHSPA12B is neglectable in volunteers and patients with SIRS and sepsis, but much higher in most patients with severe sepsis. For a power of 0.9 and α = 0.05, 30 patients is required to detect a ratio of 80% in severe sepsis group higher than control groups, and the sample size in the present study is adequate for such a statistical requirement. Patient in the control group is another problem of this case-control study. We did not included a cohort of patients with organ dysfunction induced by non-infectious SIRS. Thus we did not know if eHSPA12B is also predictive to prognosis of other critical illness. The diagnostic and prognostic value should be further investigated in a larger-size clinical trial.

In conclusion, our present study revealed that eHSPA12B is upregulated in plasma of both septic mice and patients. It may help us to identify the septic patients with poor outcome.

## References

[1] Martin GS, Mannino DM, Eaton S, Moss M (2003) The epidemiology of sepsis in the United States from 1979 through 2000. N Engl J Med 348: 1546–1554. 12700374 10.1056/NEJMoa022139

[2] Brun-Buisson C, Meshaka P, Pinton P, Vallet B (2004) EPISEPSIS Study Group (2004) EPISEPSIS: a reappraisal of the epidemiology and outcome of severe sepsis in French intensive care units. Intensive Care Med 30: 580–588. 14997295 10.1007/s00134-003-2121-4

[3] Engel C, Brunkhorst FM, Bone HG, Brunkhorst R, Gerlach H, et al (2007) Epidemiology of sepsis in Germany: results from a national prospective multicenter study. Intensive Care Med 33: 606–618. 17323051 10.1007/s00134-006-0517-7

[4] Sands KE, Bates DW, Lanken PN, Graman PS, Hibberd PL, et al (1997) Epidemiology of sepsis syndrome in 8 academic medical centers. JAMA 278: 234–240. 9218672

[5] Harrison DA, Welch CA, Eddleston JM (2006) The epidemiology of severe sepsis in England, Wales and Northern Ireland, 1996 to 2004: secondary analysis of a high quality clinical database, the ICNARC Case Mix Programme Database. Crit Care 10: R42. 16542492 10.1186/cc4854 PMC1550902

[6] Seymour CW, Iwashyna TJ, Cooke CR, Hough CL, Martin GS (2010) Marital status and the epidemiology and outcomes of sepsis. Chest 137: 1289–1296. 20173054 10.1378/chest.09-2661 PMC2881630

[7] Rivers E, Nguyen B, Havstad S, Ressler J, Muzzin A, et al (2001) Early goal-directed therapy in the treatment of severe sepsis and septic shock. N Engl J Med 345: 1368–1377. 11794169 10.1056/NEJMoa010307

[8] Dellinger RP, Levy MM, Rhodes A, Annane D, Gerlach H, et al (2013) Surviving Sepsis Campaign: international guidelines for management of severe sepsis and septic shock, 2012. Crit Care Med 41: 580–637. 23353941 10.1097/CCM.0b013e31827e83af

[9] Jacob ST, Banura P, Baeten JM, Moore CC, Meya D, et al (2012) The impact of early monitored management on survival in hospitalized adult Ugandan patients with severe sepsis: a prospective intervention study*. Crit Care Med 40: 2050–2058. 22564958 10.1097/CCM.0b013e31824e65d7 PMC3378757

[10] Rivers EP, Jaehne AK, Nguyen HB, Papamatheakis DG, Singer D, et al (2013) Early biomarker activity in severe sepsis and septic shock and a contemporary review of immunotherapy trials: not a time to give up, but to give it earlier. Shock 39: 127–137. 23324881 10.1097/SHK.0b013e31827dafa7

[11] Gibot S, Béné MC, Noel R, Massin F, Guy J, et al (2012) Combination biomarkers to diagnose sepsis in the critically ill patient. Am J Respir Crit Care Med 186: 65–71. 22538802 10.1164/rccm.201201-0037OC

[12] Ware LB, Eisner MD, Thompson BT, Parsons PE, Matthay MA (2004) Significance of von Willebrand factor in septic and nonseptic patients with acute lung injury. Am J Respir Crit Care Med 170: 766–772. 15201135 10.1164/rccm.200310-1434OC

[13] Skibsted S, Jones AE, Puskarich MA, Arnold R, Sherwin R, et al (2013) Biomarkers of Endothelial Cell Activation in Early Sepsis. Shock 39: 427–432. 23524845 10.1097/SHK.0b013e3182903f0d PMC3670087

[14] Ricciuto DR, dos Santos CC, Hawkes M, Toltl LJ, Conroy AL, et al (2011) Angiopoietin-1 and angiopoietin-2 as clinically informative prognostic biomarkers of morbidity and mortality in severe sepsis. Crit Care Med 39: 702–710. 21242795 10.1097/CCM.0b013e318206d285

[15] Aird WC (2003) The role of the endothelium in severe sepsis and multiple organ dysfunction syndrome. Blood 101: 3765–3777. 12543869 10.1182/blood-2002-06-1887

[16] Guitton C, Gérard N, Sébille V, Bretonnière C, Zambon O, et al (2011) Early rise in circulating endothelial protein C receptor correlates with poor outcome in severe sepsis. Intensive Care Med 37: 950–956. 21394629 10.1007/s00134-011-2171-y PMC3529933

[17] Han Z, Truong QA, Park S, Breslow JL (2003) Two Hsp70 family members expressed in atherosclerotic lesions. Proc Natl Acad Sci U S A 100: 1256–1261. 12552099 10.1073/pnas.252764399 PMC298760

[18] Hu G, Tang J, Zhang B, Lin Y, Hanai J, et al (2006) A novel endothelial-specific heat shock protein HspA12B is required in both zebrafish development and endothelial functions in vitro. J Cell Sci 119: 4117–4126. 16968741 10.1242/jcs.03179

[19] Zhou H, Qian J, Li C, Li J, Zhang X, et al (2011) Attenuation of cardiac dysfunction by HSPA12B in endotoxin-induced sepsis in mice through a PI3K-dependent mechanism. Cardiovasc Res 89: 109–118. 20733008 10.1093/cvr/cvq268

[20] Ma Y, Lu C, Li C, Li R, Zhang Y, et al (2013) Overexpression of HSPA12B protects against cerebral ischemia/reperfusion injury via a PI3K/Akt-dependent mechanism. Biochim Biophys Acta 1832: 57–66. 23046810 10.1016/j.bbadis.2012.10.003

[21] Yeh CH, Tseng R, Zhang Z, Cortes J, O’Brien S, et al (2009) Circulating heat shock protein 70 and progression in patients with chronic myeloid leukemia. Leuk Res 33: 212–217. 18715642 10.1016/j.leukres.2008.07.012 PMC4163801

[22] Ogawa K, Kim HK, Shimizu T, Abe S, Shiga Y, et al (2012) Plasma heat shock protein 72 as a biomarker of sarcopenia in elderly people. Cell Stress Chaperones 17: 349–359. 22144131 10.1007/s12192-011-0310-6 PMC3312957

[23] Zhu J, Wang J, Sheng Y, Zou Y, Bo L, et al (2012) Baicalin improves survival in a murine model of polymicrobial sepsis via suppressing inflammatory response and lymphocyte apoptosis. PLoS One 2012 7: e35523. 10.1371/journal.pone.0035523 PMC3348138 22590504

[24] Levy MM, Fink MP, Marshall JC, Abraham E, Angus D, et al (2003) International Sepsis Definitions Conference: 2001 SCCM/ESICM/ACCP/ATS/SIS International Sepsis Definitions Conference. Crit Care Med 31: 1250–1256. 12682500 10.1097/01.CCM.0000050454.01978.3B

[25] Manley MO, O’Riordan MA, Levine AD, Latifi SQ (2005) Interleukin 10 extends the effectiveness of standard therapy during late sepsis with serum interleukin 6 levels predicting outcome. Shock 23: 521–526. 15897804

[26] Cheng B, Xie G, Yao S, Wu X, Guo Q, et al (2007) Epidemiology of severe sepsis in critically ill surgical patients in ten university hospitals in China. Crit Care Med 35: 2538–2546. 17828034 10.1097/01.CCM.0000284492.30800.00

[27] Lefrant JY, Muller L, Raillard A, Jung B, Beaudroit L, et al (2010) Reduction of the severe sepsis or septic shock associated mortality by reinforcement of the recommendations bundle: a multicenter study. Ann Fr Anesth Reanim 29: 621–628. 20634026 10.1016/j.annfar.2010.04.007

[28] Suarez D, Ferrer R, Artigas A, Azkarate I, Garnacho-Montero J, et al (2011) Cost-effectiveness of the Surviving Sepsis Campaign protocol for severe sepsis: a prospective nation-wide study in Spain. Intensive Care Med 37: 444–452. 21152895 10.1007/s00134-010-2102-3

[29] Levy MM, Artigas A, Phillips GS, Rhodes A, Beale R, et al (2012) Outcomes of the Surviving Sepsis Campaign in intensive care units in the USA and Europe: a prospective cohort study. Lancet Infect Dis 12: 919–924. 23103175 10.1016/S1473-3099(12)70239-6

[30] Cardoso T, Carneiro AH, Ribeiro O, Teixeira-Pinto A, Costa-Pereira A (2010) Reducing mortality in severe sepsis with the implementation of a core 6-hour bundle: results from the Portuguese community-acquired sepsis study (SACiUCI study). Crit Care 14: R83. 20459716 10.1186/cc9008 PMC2911711

[31] Wang JF, Yu ML, Yu G, Bian JJ, Deng XM, et al (2010) Serum miR-146a and miR-223 as potential new biomarkers for sepsis. Biochem Biophys Res Commun 394: 184–188. 20188071 10.1016/j.bbrc.2010.02.145

[32] Gouel-Chéron A, Allaouchiche B, Guignant C, Davin F, Floccard B, et al (2012) Early interleukin-6 and slope of monocyte human leukocyte antigen-DR: a powerful association to predict the development of sepsis after major trauma. PLoS One 7: e33095. 22431998 10.1371/journal.pone.0033095 PMC3303782

[33] Heper Y, Akalin EH, Mistik R, Akgöz S, Töre O, et al (2006) Evaluation of serum C-reactive protein, procalcitonin, tumor necrosis factor alpha, and interleukin-10 levels as diagnostic and prognostic parameters in patients with community-acquired sepsis, severe sepsis, and septic shock. Eur J Clin Microbiol Infect Dis 25: 481–491. 16896829 10.1007/s10096-006-0168-1

[34] Miguel-Bayarri V, Casanoves-Laparra EB, Pallás-Beneyto L, Sancho-Chinesta S, Martín-Osorio LF, et al (2012) Prognostic value of the biomarkers procalcitonin, interleukin-6 and C-reactive protein in severe sepsis. Med Intensiva 36: 556–562. 22495097 10.1016/j.medin.2012.01.014

[35] Rose WE, Eickhoff JC, Shukla SK, Pantrangi M, Rooijakkers S, et al (2012) Elevated serum interleukin-10 at time of hospital admission is predictive of mortality in patients with Staphylococcus aureus bacteremia. J Infect Dis 206: 1604–1611. 22966128 10.1093/infdis/jis552 PMC6281403

[36] Walkey AJ, Wiener RS, Ghobrial JM, Curtis LH, Benjamin EJ (2011) Incident stroke and mortality associated with new-onset atrial fibrillation in patients hospitalized with severe sepsis. JAMA 306: 2248–2254. 22081378 10.1001/jama.2011.1615 PMC3408087

[37] Walkey AJ, Greiner MA, Heckbert SR, Jensen PN, Piccini JP, et al (2013) Atrial fibrillation among Medicare beneficiaries hospitalized with sepsis: incidence and risk factors. Am Heart J 165: 949–955. 23708166 10.1016/j.ahj.2013.03.020 PMC3695631

[38] Wang HE, Shapiro NI, Griffin R, Safford MM, Judd S, et al (2012) Chronic medical conditions and risk of sepsis. PLoS One 7: e48307. 23118977 10.1371/journal.pone.0048307 PMC3485139

